# COVID-19 impact on newly initiated and restarted antiretroviral treatment patients in the Eastern Cape, South Africa

**DOI:** 10.4102/phcfm.v15i1.3811

**Published:** 2023-02-16

**Authors:** Neil M. Orr, Helen Hajiyiannis, Tselisehang Motuba

**Affiliations:** 1 Department of Research, Centre for AIDS Development, Research and Evaluation (CADRE), Pretoria, South Africa; 2 Department of Research, Centre for AIDS Development, Research and Evaluation (CADRE), East London, South Africa

**Keywords:** COVID-19, ART patients, RIC, LTF, defaulting, PLHIV, South Africa, CHWs

## Abstract

**Background:**

Initiating newly diagnosed people living with human immunodeficiency virus (HIV) onto antiretroviral treatment (ART) and retaining patients on treatment are vital to South Africa’s ART programme. In 2020, coronavirus disease 2019 (COVID-19) and its accompanying containment (lockdown) measures presented unprecedented challenges to achieving these objectives.

**Aim:**

This study describes the impact of COVID-19 and related restrictions on district-level numbers of newly diagnosed people living with HIV and defaulting ART patients.

**Setting:**

Buffalo City Metropolitan Municipality (BCMM) in the Eastern Cape of South Africa.

**Methods:**

Mixed-methods approach: Monthly aggregated electronic patient data (newly initiated and restarted on ART) from 113 public healthcare (PHC) facilities were analysed (December 2019 to November 2020) across varying levels of COVID-19 lockdown regulation periods; telephonic in-depth interviews at 10 rural BCMM PHC facilities were conducted with facility staff, community health workers (CHWs) and intervention personnel.

**Results:**

The number of newly initiated ART patients decreased dramatically compared with pre-COVID-19 levels. The overall number of restarted ART patients increased in response to fears of co-infection with COVID-19. Facility-level communications and community outreach promoting HIV testing and treatment were disrupted. Novel approaches to providing services to ART patients were developed.

**Conclusion:**

Programmes for identifying undiagnosed people living with HIV and services aimed at retaining ART patients in care were profoundly impacted by COVID-19. The value of CHWs was highlighted, as were communication innovations.

**Contribution:**

This study describes the impact of COVID-19 and related regulations on HIV testing, ART initiation and adherence to treatment in a District of the Eastern Cape of South Africa.

## Introduction

South Africa’s antiretroviral treatment (ART) programme for people living with human immunodeficiency virus (HIV) is the largest ART programme globally.^1^ Approximately 8.2 million people were living with HIV in 2021, reflecting an HIV prevalence of 13.7%.^2^ Estimates of the percentage of people living with HIV initiated and considered retained in care (RIC) on ART in South Africa vary from 66.9% to 72%.^3,4^ Of those people living with HIV RIC, 91% achieved a suppressed viral load in 2020,^4^ and the number of people dying from acquired immunodeficiency syndrome (AIDS) decreased from 181 497 (30.4% of all deaths) in 2002 to 85 154 (12.2% of all deaths) in 2021 as a direct result of ART availability.^2^

A serious challenge to achieving and sustaining the benefits of RIC on ART is defaulting. Defaulting consists of two groups: those who interrupt ART and subsequently return to treatment within 89 days (referred to as early missing and late missing, if the person living with HIV on ART misses a scheduled appointment for 2–3 weeks, or 2 weeks to 89 days, respectively) and those who do not return for 90 days or longer (lost to follow-up; [LTF]).^5,6^ Loss to follow-up in particular reduces the immunological benefits of ART and may result in increased treatment failure, risk of opportunistic infections, viral load increase, Clusters of differentiation 4 (CD4) cell decline, drug resistance and increased mortality.^7,8^

Causes of treatment interruption and LTF in countries in sub-Saharan Africa include poor nutritional status, transport costs, waiting time at health facilities, family pressure, stigma, time needed to access treatment, the cost of remaining on treatment, the relative health of the patient, lower CD4 count, tuberculosis (TB) co-infection, adverse drug reactions, younger age, poor access to services, relocation to a different area and gaps in service delivery such as poor follow-up of patients. Health facilities with larger numbers of ART patients are less likely to actively trace patients who do not return for appointments because of high workloads.^8,9,10^

In 2020, the coronavirus disease 2019 (COVID-19) pandemic and its accompanying containment measures added another challenge to health services and to retention in care for those on ART. Coronavirus disease 2019 was first identified in the Wuhan province of China in December 2019 and is caused by the severe acute respiratory syndrome coronavirus 2 (SARS-CoV-2).^11^ In South Africa, the first wave (a time period consisting of an increase in infections, a peak of infections, followed by a decline to pre-wave levels) of COVID-19 infection commenced in March 2020 and extended to November 2020. During this time, 790 004 cases were reported in South Africa, with 21 535 deaths. During the last month of the first wave (November 2020), the Eastern Cape province of South Africa had between 50% and 55% of the country’s positive cases and was accounting for more than half of the country’s deaths from COVID-19.^12^

In order to contain the spread and impact of the disease, the South African government introduced a range of regulations. These were based on the declaration of a national disaster on 15 March 2020.^13^ On 23 March 2020, the first national lockdown (Level 5) was announced, which commenced on 27 March 2020.^14^ The five-level lockdown system^15^ describes a range of precautions and restrictions pertaining to all civil society sectors and industries, private and public health services, transportation, emergency services, supply chains, public administration services, education and personal movement. At all lockdown levels, the utilisation of personal protective equipment (PPE; e.g. cloth face masks), hand sanitation and hygiene and social distancing were compulsory. Medical, social work, counselling and relief services were permitted at all lockdown levels. At Levels 4 and 5, the public was required to stay at home, except for essential services (including child immunisation, pre- and postnatal maternal health, sexual and reproductive health and HIV services) and to obtain essential goods. Public transport was restricted to providing and accessing these essential services. Interprovincial travel was only permitted at Levels 1 and 2, and the general population was only permitted to go to work in Levels 1–3 if social distancing could be guaranteed at the place of work.

The COVID-19 pandemic and the accompanying containment regulations were unprecedented in modern times. The aim of the study was to describe the immediate and short-term impact of COVID-19 and the associated regulations from the start of the COVID-19 pandemic (March 2020) to November 2020 on the number of ART patients newly initiated on ART and those LTF ART patients who restarted ART in Buffalo City Metropolitan Municipality (BCMM) in the Eastern Cape province of South Africa.

## Research methods and design

### Study design

The study utilised a mixed-methods approach, with quantitative data being the primary data source and qualitative data as supplementary.

### Setting

Buffalo City Metropolitan Municipality is a Category A municipality in the Eastern Cape province of South Africa, and includes metropolitan urban areas, small towns, informal settlements and rural areas, with a projected population of 929 000 in 2021.^16^ In 2019, BCMM had 113 public healthcare (PHC) facilities, including 82 clinics, seven community health facilities, six hospitals and 18 mobile clinics.^17^

### Study population and sampling design

The researchers conducted an evaluation of a patient records intervention implemented by the Small Projects Foundation (SPF) at 10 rural PHCs in BCMM that commenced in June 2019 and terminated in November 2020. Public healthcare facilities were nominated by the Department of Health (DOH) to participate in the intervention on the basis of having relatively low patient burdens and not participating in similar interventions. Both quantitative and qualitative data described are derived from this evaluation.

All facility managers, ART nurses, administrative clerks, DOH community health workers (CHWs), SPF CHWs, SPF intervention programme managers and coordinators were invited to voluntarily participate in the study. Written informed consent was obtained from those who participated.

### Data collection

Quantitative data were obtained from the BCMM District DOH’s TIER.net system, an electronic patient management system for all HIV and TB patients used by the DOH across all PHC facilities in South Africa. Aggregated TIER.net data were obtained for ART patients newly initiated and those restarted on treatment at all 113 BCMM PHC facilities from December 2019 to November 2020.

Semistructured telephonic in-depth individual interviews were conducted with 30 PHC facility personnel (six facility managers, one facility manager and ART nurse, three ART nurses, six administration clerks, two DOH CHWs and 12 CHWs sponsored by the SPF) at 10 rural PHCs in BCMM and six SPF intervention personnel.

The interviews probed the impact of COVID-19 on identifying and responding to early missing, late missing and LTF ART patients, identified from facility TIER.net reports.

### Data analysis

The total monthly aggregated TIER.net data across all 113 PHC facilities in BCMM for people newly initiated on ART and LTF patients restarted on ART were collated and described for December 2019 until November 2020. These monthly totals were graphically described across the various levels of COVID-19 lockdown levels.

Telephonic interviews were audio-recorded, translated, transcribed verbatim and coded thematically using HyperRESEARCH 4.5.1 (Researchware, Inc., Randolph, Massachusetts, United States).

### Ethical considerations

The study received ethical clearance from the South African Human Sciences Research Council (reference number REC 04/19/02/20) and the Eastern Cape DOH Research Committee (reference number EC-202009-023).

Only PHC clinics that participated in the SPF intervention were eligible for participation in the evaluation. Permission was obtained from the facility manager at all 10 intervention PHCs for facility personnel to participate. Permission for accessing anonymous aggregated TIER.net data was obtained from the BCMM DOH, under the terms of a memorandum of understanding with the SPF concerning the intervention.

## Results

### Quantitative data analysis results

The analysis of aggregated TIER.net data for all health facilities in BCMM from December 2019 to November 2020 provides a broad context regarding the impact of COVID-19 and lockdown levels on the initiation onto ART of newly diagnosed people living with HIV and the recall and restarting on ART among LTF patients in BCMM facilities.

**FIGURE 1:**
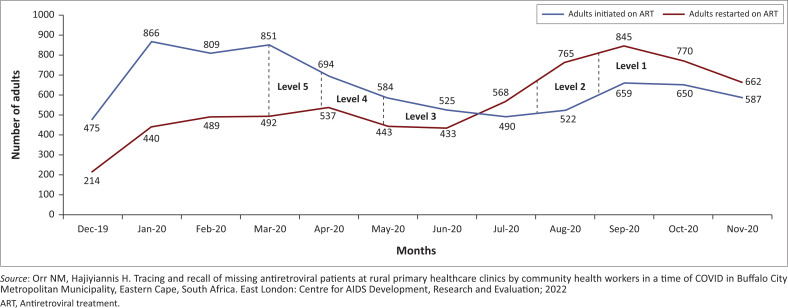
Antiretroviral treatment patients initiated and restarted in Buffalo City Metropolitan Municipality (December 2019 to November 2020).

Figure 1 describes the total number of adults aged 15 years and older in BCMM who were either newly initiated on ART or LTF patients who were traced and restarted on ART, from December 2019 until November 2020.

The periods and levels of COVID-19 lockdown are also indicated over the same period. The periods of the lockdown levels were as follows: 27 March 2020 to 30 April 2020 (Level 5); 01 May 2020 to 31 May 2020 (Level 4); 01 June 2020 to 17 August 2020 (Level 3); 18 August 2020 to 20 September 2020 (Level 2); and 21 September 2020 to 28 December 2020 (Level 1).^19^

Commencing with the pre-COVID-2019 period, it is evident from Figure 1 that December 2019 had relatively low levels of newly initiated and restarted ART patients. This is probably because of the fact that many public and private sectors in South Africa either slow down or close over the Christmas holiday season, and many South Africans travel during the longest holidays for all school and tertiary learners.^20^

In the period from January 2020 to March 2020 the data from Figure 1 appear to suggest that efforts to identify and initiate people living with HIV on ART resumed, as well as tracking and tracing efforts to recover and restart LTF patients. The average number of newly initiated ART patients in this period was 842 (compared with 475 in December 2019), and the average number of LTF patients who recovered and restarted on ART was 473 (compared with 214 in December 2019).

The impact of the 2-month period of Levels 5 and 4 lockdown regulations is evident from Figure 1 in the sharp decrease (30.6%) from the pre-COVID-19 average of 842 to 584 at the end of May 2020 in newly initiated ART patients. During this period, all interprovincial travel was prohibited and the public was required to work from home. Essential services continued, but all community programmes – including screening and testing for HIV – ceased entirely. As indicated in Figure 1, the trend of reductions in newly initiated ART patients continued through to lockdown Level 3 (June 2020 to mid-August 2020), with a low point of 490 patients (i.e. 41.8% lower than the 3-month pre-COVID-19 average from January 2020 to March 2020) being initiated onto ART.

The number of people newly initiated onto ART appears to improve from Level 2 to Level 1, with a peak of 659 in September 2020. However, as described in Figure 1, by November 2020 the number of newly initiated ART patients remained at 30.3% below the average pre-COVID-19 levels.

According to clinic personnel interviewed, clinics were frequently closed for short periods of time because of staff becoming infected with COVID-19. During lockdown Levels 5, 4 and 3, CHWs were not permitted to do home visits to conduct community tracking and tracing and instead conducted telephonic tracing and tracking. Restrictions on CHWs travelling into communities – often to support COVID-19 contact tracing – eased from September 2020 when the country transitioned to Level 2 lockdown. It is apparent that the return of CHWs to community activities is associated with an increase in initiation of newly diagnosed people living with HIV onto ART.

The recovery and restarting of LTF patients on ART for the first 3 months of COVID-19 lockdown appears unchanged (average 471 from April to June 2020) compared with the 3-month pre-COVID-19 period (average 473). However, according to Figure 1, from July 2020 the number of recovered and restarted LTF patients rose sharply, to peak at 845 in September 2020. This represents an increase of 79% from both the 3-month pre-COVID-19 period and the first 3 months of the COVID-19 lockdown period. It is notable that the number of LTF patients who recovered and restarted on ART exceeded those newly initiated on ART from July 2020 (Level 3). This reversal in trends remained until November 2020. From July 2020 to November 2020, those LTF patients recovered and restarted on ART averaged 772 per month, compared with an average of 582 for newly initiated ART patients.

### Qualitative analysis results

The 30 participants from 10 rural PHC facilities within BCMM and six SPF intervention personnel provided detailed personal observations regarding the impact of COVID-19 and accompanying lockdown regulations. These are presented according to the population groups affected, commencing with people living with HIV on ART.

The reaction of people living with HIV on ART – whether adherent or defaulting – appeared mixed: many ART (and other chronic) patients were ‘afraid to come to the clinic because they are afraid of COVID’ (CHW, Facility 3, 26 February 2021). However, according to some facility personnel, COVID-19 had a beneficial impact on ART defaulting rates:

‘They were scared … a person knows that okay, I am HIV positive and my immune system is compromised. So, I’m now not taking the treatment for some time, maybe a year or a few months. So, most of the clients that were HIV positive were really afraid of COVID, so they knew to come to the clinic so that they can take the treatment. So, most of our defaulters did come back.’ (ART nurse, Facility 7, 21 February 2021)

The source of the anxiety of defaulting ART patients regarding co-infection with HIV and COVID-19 was described as follows: ‘It’s because on the news, it’s been saying if you are not taking your treatment … it’s going to be bad on you’ (Administration clerk, Facility 7, 29 January 2021).

However, it was considered uncertain whether the fear of COVID-19 would serve as a sustainable motivation for LTF patients to remain on ART in the medium and long term: ‘I don’t know if maybe now they stopped being scared or they are still taking treatment. I am not sure’ (Facility manager, Facility 5, 28 January 2021).

Although it appears that COVID-19 anxieties caused LTF ART patients to seek treatment, it appears that early missing and late missing ART patients were negatively impacted by COVID-19 measures. Initially, the clinic management took steps to reduce the number of clients visiting the clinic, in order to reduce the risks of becoming infected with COVID-19 because of proximity to other people. In particular, the elderly and those who were stable on treatment (including ART) were identified, and CHWs took their medications to their homes. This was purportedly in addition to DOH programmes providing medication collection at locations (pick-up points) external to health facilities for registered stable patients:

‘During this COVID … we wanted to decrease the number of clients visiting the clinic … We retrieve the client’s files and see okay, somebody was supposed to come here … we only bring those that have chronic diseases that are not controlled, we only call those. The others we deliver their packets to their homes.’ (Facility manager, Facility 7, 03 February 2021)

According to CHWs from two facilities, this system worked well in reducing levels of missing and LTF ART patients, although the programme was directed at all chronic patients, not only at those who had defaulted on ART. Also, these deliveries did not replace standard blood tests and check-ups. However, as the numbers of COVID-19 infections increased, CHWs were stopped from doing these home deliveries because of the risk of COVID-19 infection. An ART nurse reported that after home deliveries of medications by CHWs to ART patients were stopped as a result of COVID-19 risks, CHWs telephoned the ART patients to collect their medications at the facility and also to come for blood tests. As a result, the levels of early missing and late missing ART patients increased, as these patients were waiting for their medications to be delivered. Some received calls to collect their medications from the facility, but some did not receive such calls and did not collect such medications (ART nurse, Facility 6, 03 February 2021).

According to a facility manager, there were also times when the mobile clinic did not conduct routine services to remote villages because of either COVID-19, staff shortages caused by COVID-19 or the mobile clinic needing to be serviced. At this clinic, there was apparently an increase in ART defaulters as a result, specifically from April 2020 to September 2020 (i.e. Levels 5 to 2). During this period, the clinic relied on the CHWs to phone people on ART to collect their medications themselves from the facility. The situation with the mobile clinic was back to normal by January 2021 (Facility manager, Facility 3, 02 February 2021).

Facility personnel experienced a range of personal impacts, both directly from COVID-19 itself and from the lockdown regulations. In this regard, it is important to observe that during all levels of lockdown, PHC facilities were considered essential services and thus remained open. Also, even in the strictest Level 5 lockdown period, the public were permitted to go to these facilities for medical care, placing these PHC facilities at the frontline of the pandemic. Facility staff were consequently permitted to travel to and from work during the initial high levels of the lockdown but only under strict guidelines, whereas nonessential services and normal public transport travel were forbidden during Levels 5 and 4 of the lockdown, making it difficult for staff to travel to facilities. It is noticed that the period under examination preceded the roll-out of COVID-19 vaccinations for healthcare workers, which commenced in February 2021.

One of the most frequently reported personal impacts of COVID-19 on facility staff concerned working in an environment where the risk of becoming infected with COVID-19 was high, which produced high levels of anxiety. According to two facility managers, staff concerns focused on several sources of risk of becoming infected with COVID-19, including during travel to and from work because they used public transport (when available), not knowing what other facility staff were doing (i.e. who they were interacting with) over weekends and also from attending funerals of those who had passed away from COVID-19. In addition, community members were being tested for COVID-19 at community centres but would go to the facility for their results. As a result, facility staff were acutely aware of interacting with COVID-19-positive patients at the facility (Facility manager, Facility 5 28 January 2021, Facility manager, Facility 8, 25 January 2021).

The anxiety of facility staff regarding becoming infected with COVID-19 appears warranted. One facility manager described how the facility lost one of its five nurses to COVID-19, and another was placed on sick leave for an extensive period of time because of COVID-19. This facility manager also described how the loss of these nurses had a serious impact upon patient services, reducing a complement of five nurses to three.

At least 3 of the 10 rural PHC facilities were reported to have been closed for periods of 1 to 10 days as a result of diagnoses of COVID-19 among staff (Facility manager, Facility 5, 28 January 2021; Facility manager, Facility 6; Facility manager, Facility 7, 03 February 2022). Some facilities were closed more than three times from March 2020 onwards, and PHC Facilities 7 and 10 were closed several times because of COVID-19 (SPF personnel).

Instances of facility staff becoming infected or isolating at home as a result of possible exposure led to staff shortages, including only having one nurse working during some periods:

‘For the whole – is it 10 months now? We’ve closed one day, our clinic. Because we waited for fumigation. But there were days where there was only one nurse working, because the others were on quarantine or isolation.’ (Facility manager, Facility 5, 28 January 2021)

It was also stated that when a facility closed for a period of time, ART (and other) patients were referred to surrounding facilities, but some could not travel the additional distances and therefore defaulted on their treatment (Facility manager, ART nurse and Administration clerk, Facility 7, 29 January 2021).

There were no reports of actual medication stock shortages. However, one facility manager stated that they were notified that the supply depot was running low on some types of antiretrovirals because of delays at the supply depot as a result of COVID-19. The facility manager added that they did, however, receive the requested medications. Also, the facility had all the medications required for patients (Facility manager, Facility 6, 27 Jnauary 2021).

According to most facility study participants, facility staff received adequate supplies of PPE throughout the study period. The exceptions were Facilities 1 and 7, where there was a shortage of protective gowns and aprons for ART nurses conducting household screening and testing. It was stated that these nurses had to use the same gown for an entire day, whereas they should have had enough to change the gown after each household screening (Administrative clerk, Facility 1; ART nurse, Facility 7, 21 February 2021).

All group gatherings – including nonessential meetings – and nonessential travel were prohibited during COVID-19. This meant that staff trainings, weekly and monthly staff meetings, travelling to subdistrict DOH offices to verify and submit TIER.net reports by administration clerks and conducting support groups ceased (Facility manager, Facility 5, 28 January 2021). An administration clerk said that regular meetings no longer occurred, but sometimes they had quick five minute meetings when there were announcements, but they needed to be quick so that patients were not kept waiting, thus increasing their risk of exposure to COVID-19 (Administration clerk, Facility 1).

An ART nurse described how she had started a support group with diabetic patients, but this had stopped because of COVID-19 (ART nurse, Facility 6, 03 February 2021). Social services were also stopped as a result of COVID-19, and this impacted community members seeking social grants. All social workers were reportedly instructed to return to their head office or work from home (Facility manager, Facility 6, 27 January 2021).

Community health workers, most of whom live in the communities they serve, were profoundly impacted, both personally and in terms of their ability to conduct community outreach, conduct home visits and do tracking and tracing of ART defaulters. When permitted to conduct home visits, several CHWs reported anxiety in conducting such visits:

‘Home visits are difficult because we don’t know what we are going to find at that house … sometimes you are going to a COVID area … Even though we have got PPE for going out … we are afraid to go to these households because we don’t know what’s happening in the households, and because COVID is real and it’s killing people … If I find the [*phone*] number of that person, I make a call to that person instead of going there. But if I don’t have the contact number, [*then*] you just have to go, and then pray on the way that God protects you.’ (CHW, Facility 1, 26 January 2021)

This CHW also said that when she gets home, she washes her clothes and hands because ‘you don’t know if that person [just visited at home] has it or not’, and ‘you don’t want to come with the illness … to your home’. The CHW added that although they were supplied with PPE, the coats could not be washed every day if they were to last a full month (CHW, Facility 1, 26 January 2021).

Another CHW stated that the ART clients visited at home were reluctant to be in contact with the CHW because of perceived higher risk of exposure to COVID-19:

‘When you go to the homes now, the people do not want to talk with you because they said at the clinic there [*are*] people that have COVID and you come with COVID-19 to us.’ (CHW, Facility 8, 26 January 2021)

Several CHWs reportedly did home visits very reluctantly, if at all, largely because of fear of contracting COVID-19, even though they were provided with PPE. Instead, the main contact with missing and LTF patients was telephonic:

‘[*At*] this COVID time we are [*only*] using telephone … [*We*] phone those people. … Before COVID we were going door-to-door but as COVID goes on, we are using telephone calls … I try and try until I get through to her. … If she or he is not answering the phone, I send her SMS and then if she does not reply to the SMS, I keep on calling until he answers the phone.’ (CHW, Facility 6, 01 February 2021)

According to some CHWs, making phone calls to ART defaulters had distinct advantages over home visits, such as reducing fears of being identified as a people living with HIV: ‘When you are talking on the phone, the defaulters are free … they don’t have a fear of the people around’ (CHW, Facility 8, 26 January 2021). Another CHW said that calling people was ‘straight’ and ‘it was easy to get them’ compared with home visits but that some could not be contacted, and home visits were required (CHW, Facility 10, 09 February 2021). The ease of communicating telephonically with traced defaulters was in contrast to several reports by CHWs concerning verbal abuse and sexual harassment when conducting home visits during tracking and tracing of ART defaulters:

‘When I got someone and talk to him, he shouts at me “don’t come here again.” Those other ones they say that “I’ll never go to the clinic again” … I think … they are embarrassed that I went to their house.’ (CHW, Facility 3, 26 February 2021)

‘My fear is … the defaulters have anger … [*For*] example, when we are distributing condoms at the community, he says “what is this?” You tell him that this is a condom, you teach and encourage him to use it. Then he says “give me the condom and teach me how to use it on you.”’ (CHW, Facility 8, 26 February 2021)

At one PHC facility, the ART nurse created social media groups for missing and LTF ART patients and communicated lists of these patients to CHWs via social media. A CHW observed that this form of communication was preferable to being at the facility, and it worked well:

‘Last year we didn’t come to the clinic … So the sister in charge created the WhatsApp group and gave us the defaulters’ list on that group and then we traced them and we reported again on the WhatsApp group … On my WhatsApp I have a group … I have those people who are on ART there, but they don’t go to the clinic. So I talked to them [*on*] WhatsApp and asked them to please go to the clinic because the sister reported that they are needed at the clinic. Please, please, so they go. I talk to them and … they know me. As soon as they come to the clinic, they say to the person who is seeing them that they were sent by me.’ (CHW, Facility 1, 26 January 2021)

Community health workers reported that the use of cellular phones for communicating with patients, staff at the facilities and their supervisors was a temporary solution, because it was only funded by the SPF for the period of time where they were not permitted to do home visits, and the CHWs could not personally sustain the airtime costs after the funding ended.

It was observed that facility staff and CHWs did not mention ethical considerations of communicating confidential patient information on such social media platforms.

In line with COVID-19 restrictions regarding group gatherings, no meetings took place between CHWs and their supervisors and no trainings occurred for most of the lockdown period.

It is unclear when CHWs returned to working from mobile clinics. Based on a statement from one facility manager (Facility manager, Facility 3, 02 February 2021) this probably only occurred after November 2020 because the clinical staff on the mobile clinics are sourced from the clinical staff at the facility, and there was a shortage of facility staff at the clinics during most of the COVID-19 lockdown levels.

## Discussion

Overall, the district-level TIER.net data clearly show that the COVID-19 pandemic and its concomitant regulations were associated with a marked decrease in the number of people being diagnosed with HIV and newly initiated onto ART and a substantial increase in the number of LTF ART patients restarted on treatment.

Compared with the 3-month pre-COVID-19 lockdown average, the strictest levels of the lockdown (Levels 5, 4 and part of Level 3) were associated with a 41.8% reduction in people living with HIV being initiated onto ART. From mid–Level 3 of lockdown, the monthly totals of people initiated onto ART increased, but never returned to pre-COVID-19 lockdown levels by the end of November 2020.

Prior to the COVID-19 lockdown, the monthly totals of LTF ART patients restarted on treatment were consistently lower than newly initiated ART patients. This remained unchanged during the first 3 months of the lockdown (Levels 5, 4 and part of Level 3). However, from July 2020, there was a dramatic increase in the number of LTF patients restarted on ART, with an overall increase of 79% compared with pre-COVID-19 lockdown levels. Furthermore, monthly totals of restarted ART patients exceeded newly initiated patients from July 2020 until the end of the study in November 2020.

The primary cause of the initial reductions in newly initiated ART patients during Levels 5, 4 and part of Level 3 of COVID-19 lockdown periods appears to be the total cessation of community HIV screening and testing activities because of limitations on the gathering of groups and restrictions on travel to limit risk of exposure and transmission of COVID-19.

As the restrictions eased, so the levels of newly initiated ART patients began to rise again, although not returning to pre-COVID-19 levels by November 2020. It is also notable that the levels of newly initiated ART patients appear linked to the mobility and activities of CHWs within communities, such as HIV education, screening and testing campaigns.

The marked increase in the return of previously LTF ART patients to restart ART is noteworthy. The levels of restarting ART rose consistently as travel and other restrictions eased, and the largely self-initiated return to clinical care was explained by facility staff as a result of external messaging concerning the dangers of being co-infected with both HIV and COVID-19. It appears that – for many LTF ART patients – perceptions of personal risk of illness and death were sufficiently impacted that they took action to restart ART. It is unclear why messaging concerning risks of contracting COVID-19 appeared more effective in motivating LTF patients to restart ART, compared with prior messaging concerning risks of developing HIV-related illnesses. It is recommended that the COVID-19 messaging is investigated to ascertain the cause of its effectiveness in motivating LTF patients to restart treatment.

One of the costs of protecting the general population from COVID-19 was to interrupt the education, screening, testing and initiation of newly diagnosed people living with HIV onto ART, for a significant period of time, although there are clear indications that such programmes were resumed after approximately six months. These data highlight the need for efforts to regain ground lost in the control and prevention of HIV. It is also clear that large-scale test-and-treat programmes depend heavily upon the availability of CHWs to reach communities in facility catchment areas.

The strength of the district-level TIER.net data is that they provide information from all 113 public health facilities in BCMM, including formal urban, suburban, informal urban and rural areas.

Limitations of the narrative data gathered in the telephonic interviews include the fact that the data were gathered only in relatively low patient burden rural PHC facilities, and therefore they do not reflect urban or high patient burden facility experiences during the same time period. For example, restrictions regarding public transport would be more problematic for patients in remote rural areas, while patients in high-density urban areas would have greater access to facilities because of proximity. Also, the PHC facilities included in the study were selected on the basis of having relatively small staff complements and therefore do not reflect facilities with larger staff complements. As such, the facilities in the study were likely to experience more marked impacts of staff absences and losses.

Furthermore, the CHWs at the facilities included those who were sponsored by the SPF to travel into communities to do tracking and tracing and to provide mother-and-child services, whereas DOH CHWs are often restricted to the facility because of budgetary constraints. Therefore, the experiences of these mobile CHWs are not necessarily representative of CHWs at all public facilities. The SPF CHWs were also sponsored with cellular phone airtime, while DOH CHWs typically rely on facility fixed-line telephone systems.

However, the narrative data provide granular insight into how COVID-19 – the disease and the regulations put in place to limit transmission – impacted day-to-day and overall functioning at rural PHC facilities and particularly on services to ART patients.

It is relevant to observe that the procedures – such as efforts to ensure that chronic patients receive medications – were not confined to these study facilities but reflect district-level decisions and efforts. In this regard, the narrative data gathered provide insight into overall district-level decision-making and responses to COVID-19 and lockdown regulations.

A central theme exposed in the narrative data concerns how facilities – including staff and CHWs – implemented a range of interventions to continue the provision of services to people living with HIV on ART and simultaneously to comply with COVID-19 regulations. The question arises regarding whether these efforts and measures can be adapted and used in post-COVID-19 facility operations to improve the efficiency and resilience of the public health system.

Based on the narrative data, initial efforts to reduce patient volumes at the facilities were accompanied by home delivery of medications by CHWs to all stable chronic patients. The strategy appeared to be successful. However, as COVID-19 infection rates increased, CHWs were prohibited from travelling to patients’ homes, and such deliveries ceased. Despite changing to a telephonic recall and reminder system for the collection of medications, the reported levels of early and late missing ART patients consequently increased.

In a post-COVID-19 era, the utilisation of CHWs to deliver chronic medications to patient’s homes – particularly in remote rural areas – may bear favourable consideration, as this reduces the travel and other costs of collecting medications for all chronic patients. However, as with most community outreach efforts, this requires an adequate cadre of CHWs to implement.

Notably, no medication shortages were reported, indicating a stable and functioning essential medication stock control system. The PPE – although limited – was also reported to be distributed to all facility personnel and CHWs. It is noteworthy that clinic closures were brief, although frequent in some cases. The reports regarding medication and PPE supplies, as well as rapid returns to providing health services at the facilities, indicate resilience within these areas of PHC.

The emotional impact of COVID-19 on PHC facility staff was profound: staff shortages were reported at most facilities because of death, illness and quarantine of infected staff, as well as for staff taking leave to attend funerals. Prior to COVID-19 vaccinations (which commenced three months after the termination of the study), staff were placed under great stress not only because of concerns for their personal risk of contracting COVID-19 but also regarding contracting COVID-19 while travelling to and from work, or at work, and then infecting family members at home. Despite these anxieties, there were no reports of facility personnel refusing to work at the facilities.

In conjunction with these marked increases in personal work-related anxieties, face-to-face communications between staff, CHWs and the community were severely curtailed. The marked reduction of face-to-face interpersonal communications was a major theme reported throughout all interviews, particularly concerning communication between facility staff, CHWs and ART patients.

To compensate for the restrictions in face-to-face communications, facility staff and CHWs shifted their primary medium of communication to cellular phones. As a result of apparent budgetary and network constraints, cellular phone communications utilised (although not exclusively) short message services (SMSs) and social media platforms to maintain contact between facility staff, patients and CHWs. Video communication was not mentioned because of network signal strengths and the cost of data for such purposes.

Two main issues regarding the use of cellular phones and social media platforms merit further attention: the benefits reported (versus home visits) and the ethical considerations of utilising such media to convey confidential patient information.

Facility staff and CHWs reported several benefits of cellular phone SMSs and social media – versus home visits – such as eliminating transportation costs for CHWs, being less physically strenuous in terms of accessing remote and rough terrain, being more acceptable to LTF ART patients because of greater anonymity and convenience, avoiding verbal abuse and sexual harassment during tracking and tracing of defaulters and the ability to conduct group discussions without needing to travel to a central location.

However, it was also stated that telephonic communication was not effective when the patient could not be reached telephonically, either because of the absence of network coverage, not answering the call, loss of the telephone, changes in telephone numbers or because the person (CHW or patient) could not afford the airtime or data charges. The cost of airtime and data was cited by most CHWs as a primary consideration in such communications.

Given these advantages and disadvantages, the reported results in tracking and tracing early missing, late missing and LTF ART patients, as well as maintaining contact with support group members during the COVID-19 lockdown period, suggests that telephonic and social media communications are viable options, especially with LTF ART patients who are reluctant to receive physical home visits.

Notably, there was no mention by any of the facility staff or CHWs of the ethics of communicating patient identification and contact details via SMS or via social media platforms. However, this issue was not probed during the interviews and conclusions cannot be made in this regard.

The reported benefits of utilising digital communication bears further consideration because they appear to be accessible to most – but not all – facility staff, CHWs and ART patients. Despite network and budgetary limitations, most participants in the study said that they had a cellular phone. It was also stated that telecommunication was not suitable in cases where the person had moved, had changed phone numbers or where there was no phone number on record. Thus, a mixed approach of telephonic communication, followed by home visits where needed, is suggested. This also suggests that patient registration should include emphasis on obtaining both telephonic and physical address details and encouraging registered patients to update these contact details at each facility visit or when moving to a different location.

Overall, it appears that the use of digital communications within the PHC health system requires closer attention with a view to eliminating travel costs and risks to outreach team members and providing a reliable form of communication between facilities and community members. However, the ethics of such systems need to be scrutinised to ensure that patient confidentiality is not compromised by expediency.

## Conclusion

A significant detrimental impact of the varying levels of COVID-19-related lockdown restrictions was observed on HIV testing and initiation onto ART for those who were newly diagnosed. Public healthcare facilities experienced losses of trained personnel and interruption of services. However, the unprecedented nature of this crisis produced innovation in areas such as communication mediums and highlighted the resilience of all healthcare workers. The unexpected increase in returning to treatment of LTF people living with HIV, reportedly as a result of COVID-19 health messaging, also opens new avenues to reduce defaulting of ART patients through more effective health messaging.
